# Comparison of topical purslane & topical 0.1% triamcinolone acetonide in the management of oral lichen planus - a double blinded clinical trial

**DOI:** 10.1186/s12903-023-03385-1

**Published:** 2023-09-19

**Authors:** Arul Jothi Murugan, Anuradha Ganesan, Yesoda K. Aniyan, Krithika Chandrasekar Lakshmi, Kannan Asokan

**Affiliations:** Department of Oral Medicine and Radiology, SRM Dental College, Ramapuram, Chennai, 600089 Tamil Nadu India

**Keywords:** Antioxidant, Clinical improvement, Oral lichen planus, Steroids, Topical purslane

## Abstract

**Aim and background:**

Oral lichen planus (OLP) is a chronic autoimmune mucocutaneous disorder of unknown etiology and treatment is targeted at alleviating symptoms. At present, corticosteroids are the mainstay treatment, and their side effects hamper their long-term use, demanding alternative therapy. This study intended to assess the efficacy of topical purslane *(Portulaca oleracea)* at two concentrations, 5% and 10%, in OLP and to compare the level of clinical improvement in comparison to topical 0.1% triamcinolone acetonide gel.

**Materials and methods:**

After sample size determination, thirty-four subjects confirmed histopathologically with OLP were included in the study. They were divided into 3 groups, Group 1(Control) was treated with 0.1% triamcinolone acetonide, and Group 2(Case) and 3 (Case) were treated with topical purslane 5% and 10%, respectively. They were examined at baseline, 14 days, 30 days, 60 days, and 90 days. Clinical improvement was then analyzed at the end of 90 days using a visual analog scale (VAS) and Thongprasom’s criteria.

**Results:**

The study was analyzed statistically and a *P* value of < 0.05 was considered statistically significant. Intragroup comparison revealed a statistically significant difference between the five time periods (baseline, 14 days, 30 days, 60 days, and 90 days) for the study variables (burning sensation, pain, lesion score, clinical response, symptomatic response) for all three groups (*p* = 0.001). Alleviation of all symptoms and remission of the lesion were noted for all three groups at the end of three months.

**Conclusion:**

Purslane is a magical herb with a plethora of rich nutrients, ease in accessibility and devoid of side effects. It was concluded that its preparation is beneficial and can be a safer alternative long-term drug for the management of OLP.

**Clinical significance:**

With available literature evidence, our present study is the first of its kind to formulate a topical gel with purslane to treat symptomatic OLP. Our study had a longer follow-up of 3 months compared to other studies in the literature.

## Introduction

The World Health Organization Collaborating Centre for Oral Cancer Workshop held in the United Kingdom (2020) confirmed oral lichen planus (OLP) as a potentially malignant disorder. OLP is defined as a “chronic inflammatory disorder of unknown etiology with characteristic relapses and remissions, displaying white reticular lesions, accompanied or not by atrophic, erosive, and ulcerative and/or plaque-type areas’. Lesions are frequently bilaterally symmetrical. Desquamative gingivitis may be a feature” [1]. The sites of presentation include the skin, scalp, nails, and mucous membranes of the oral and genital systems [2, 3]. Its prevalence is common in middle-age; with a female to male ratio of 1.4:1 [4].

To date, the etiology remains ambiguous, and multifactorial facets have been attributed, including genetic background, infectious agents, autoimmunity, psychological stress, and deleterious habits such as cigarette smoking and betelnut chewing. Various systemic disorders have also been hypothesized to contribute to etiology [3].

OLP has six clinical subtypes: reticular, papular, plaque - like, bullous, erythematous, and ulcerative [4]. The prevalence and incidence rates of OLP vary from 0.5% to 2.5% and 0.1% to 4.0 per 100 people, respectively, globally [5]. In the manner of its presentation, lesions in the majority are asymptomatic and painless. However, erosive, and atrophic subtypes induce pain, a burning sensation, and sensitivity to all oral functions. This in turn irrevocably affects the quality of life [2]. It manifests commonly on the buccal mucosa, palate, and tongue in the oral cavity [4]. The malignant transformation rate is 1- 2.2%. The plaque-like and erosive types are potentiated for malignant transformation [4, 6]. Considering the high risk of malignant transformation, the erosive type of oral lichen planus was excluded from the study. Reticular type of OLP was included.

Considering the autoimmune nature of the disorder, OLP is recalcitrant, and its management is oriented toward symptom alleviation**.** Corticosteroids, in topical and systemic forms, are the gold standard. Although remarkable in symptom control, they have substantial adverse effects curtailing their long-term use [7].

Phytomedicine is rich in antioxidant and anti-inflammatory characteristics, making it an excellent alternative for safer, affordable, and efficient medication [8, 9]. An in-depth literature review by Pourshahidi et al., mentions herbal preparations from Curcumin, Purslane, Aloe vera, Grape vine, liquorice, Calendula, Quercetin, Honey, Tripterygium, Paeony, Lycopene, Ignatia, Chamomile, etc., to be effective in treating OLP [10].

Purslane is an edible herb with medicinal properties. It boasts phytochemical richness, namely, flavonoids, alkaloids, coumarins, anthraquinone glycoside, cardiac glycoside, fatty acids, terpenoids, polysaccharides, vitamins, sterols, proteins, and minerals. Additionally, it has higher β-carotene, ascorbic acid, and alpha-linolenic acid levels. The pharmacological actions are myriad actions ranging from antibacterial, antiulcerogenic, anti-inflammatory, antioxidant, wound healing and purgative to emollient, muscle relaxant, and diuretic properties [11–13].

Studies by Bao and Chen et al. report that oxidative stress is one of the causative factors in the pathogenesis of OLP [14]. Nitrative and oxidative stresses have been proposed to participate in inflammation-mediated carcinogenesis OLP. Purslane possesses antioxidant and anti-inflammatory properties and contributes to free radical damage prevention [15].

## Aims and objectives

This study aims to assess and compare the therapeutic efficacy of topical purslane gel at two concentrations (5% and 10%) against topical triamcinolone acetonide 0.1% OLP management.

## Materials used

### Study design

Proper ethical clearance was obtained from the Institutional Review Board (SRMDC/IRB/2020/MDS/No.901), and the study was registered in the Clinical trial registry of India (CTRI/2021/09/036647) on 20/09/2021. A double blinded randomized controlled clinical trial was proposed and conducted in the Department of Oral Medicine and Radiology, SRM Dental College, Ramapuram, Chennai, for a duration of 16 months (March 2021 - July 2022). Randomization of participants into each group was carried out using computer generated sequence numbers and allocation of participants to each group was performed using the Excel RAND function.

As our study is a double blinded study, the primary researcher is blinded to the allocation of participants into the treatment group as they carry out the treatment and evaluate the outcome. The participants are blinded to the type of gel they are provided with. The allocation of the patients will be performed by a researcher who is not involved in patient evaluation.

### Study samples

Based on the Agha Hosseini et al. (2010) study [11], the sample size was determined with a power of 80% and an alpha error of 1%. Following the guidelines of the Helsinki declaration, participants who volunteered for the study and met the inclusion criteria were chosen. Thirty- four people with burning sensation and painful symptoms confirmed histologically and clinically as oral lichen planus were included in the study. Informed consent was obtained from the participants of the study. The included samples were further categorized randomly into 3 groups. Ten participants in control group 1 received topical 0.1% triamcinolone acetonide gel, while 12 participants in case groups 2 and 3 received topical 5% and 10% purslane gel, respectively.

### Inclusion criteria

- Patients clinically diagnosed with symptomatic OLP were further confirmed by histopathology.
- Patients who had not used systemic or topical glucocorticosteroids for at least the past 2 weeks.
- Patients who agreed not to use any other medication such as analgesics and anesthetics in either topical form or systemic form during the study.

### Exclusion criteria

- Patients who are not willing to be a part of the study.
- Patients with lichenoid lesions are thought to develop hypersensitivity reactions to drugs and dental materials.
- Patients on long-term glucocorticosteroid therapy.
- Pregnant and lactating mothers.
- Patients who are allergic to purslane.
- Participants had a clinical appearance of an erythematous, ulcerative, and bullous type of OLP.

## Methodology

### Preparation of purslane gel

Fresh leaves from Portulaca oleracea were collected from the local market in Tondiarpet, Chennai, Tamil Nadu washed with running water, shade dried, and powdered to granules. It was processed to obtain the ethanolic extract, which was formulated with the ora-base gel at 5% and 10% concentrations. The composition of the gel formulation is given in Table 1.

**Table 1:**
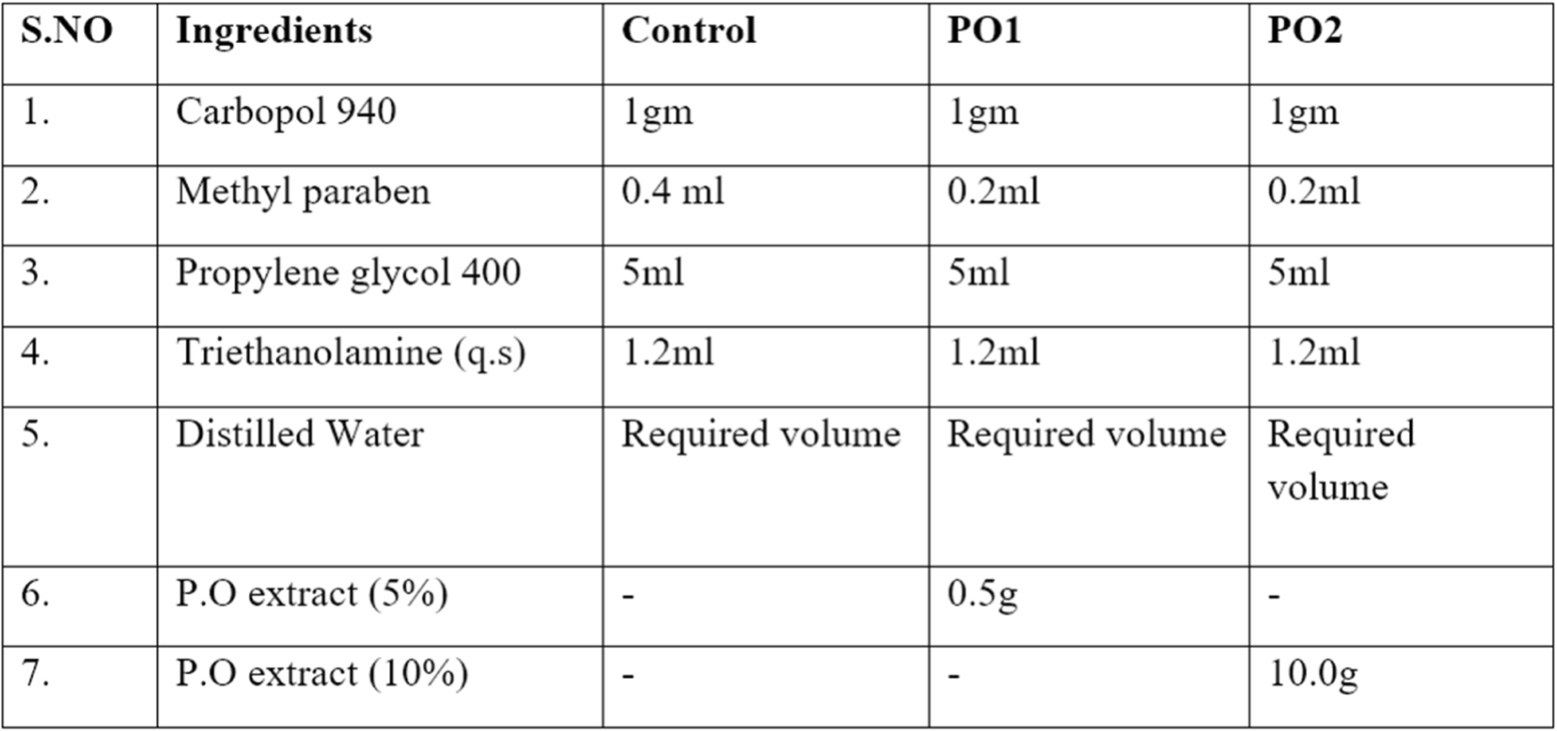
Formulation of the gel

In vitro analysis was performed for the prepared gel to evaluate antimicrobial, antioxidant and cytoprotective effects. For antimicrobial properties, a 10% concentration of purslane gel showed complete inhibition of both gram positive and gram negative bacteria. A 5% concentration of purslane gel showed no inhibitory concentration against *Escherichia coli*. The antimicrobial property of the preparation to inhibit bacterial growth is related to the synergistic effect between the active compounds of the extract. The radical scavenging activity was evaluated by free radical method that used DPPH (2,2-diphenyl-1- picryl-hydroxyl-hydrate), as an antioxidant assay. The 10% formulation showed the highest radical scavenging activity of ~ 25% - 78% and the 5% formulation showed ~ 24% - 44%. The formulations did not show cytotoxicity against the human monocyte cell line (THP-1) [16].

### Double-blinded randomized controlled trial

**Fig. 1:**
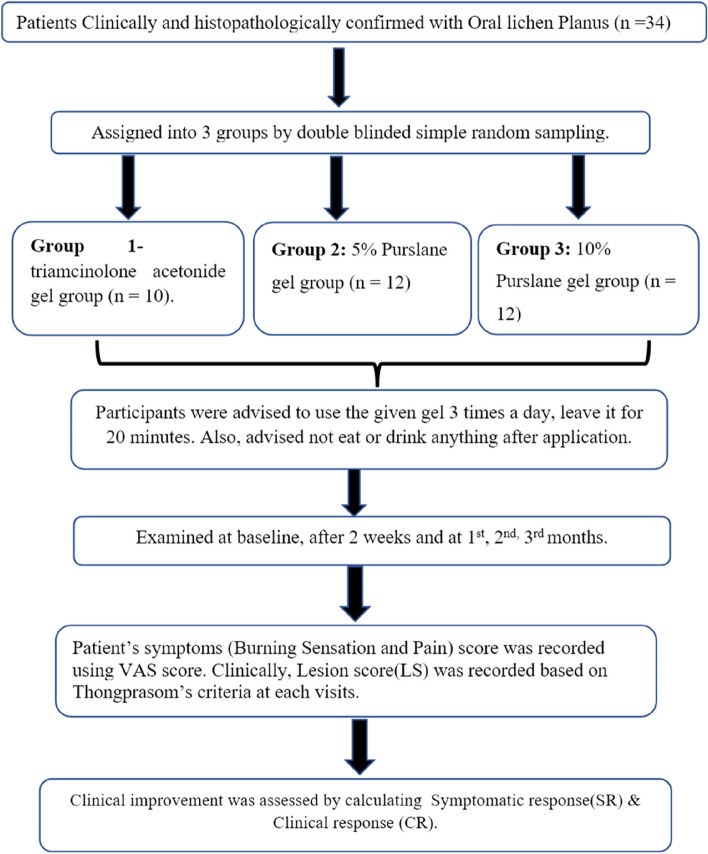
Brief protocol of the study

Participants with chief complaints of burning sensation or pain were selected for the study. Thirty-four participants with clinically diagnosed and histopathologically confirmed oral lichen planus were categorized into 3 groups by randomization. Participants were evaluated at baseline, 14 days, 30 days, 60 days, and 90 days for characteristics of burning sensation and pain scored using the Visual Analog Scale (VAS) - subjective scoring scale which has gradings from 0 - 10 where 0 is no pain, 1-3 is mild, 4–6 is moderate and 7 -10 is severe. The lesion size was scored using Thongprasom’s criteria - 0: No lesion, normal mucosa, 1: Mild white striae, no erythematous area, 2: White striae with atrophic area less than 1 cm^2^, 3: White striae with atrophic area more than 1 cm^2^, 4: White striae with ulcerative area less than 1 cm^2^, and 5: White striae with ulcerative area more than 1 cm^2^ [17]. To determine the efficacy, clinical improvement was assessed after three months by evaluating the symptomatic response (SR) and clinical response (CR). Symptomatic response (SR)—calculating the difference between the symptomatic score during the first and last visits. Clinical response (CR) - calculating the difference between the lesion scores during the first and last visits. A brief protocol of the study is shown in Fig. 1.

### Statistical analysis

The SPSS program (IBM SPSS Statistics, Version 20.0, Armonk, NY: IBM Corp.), the statistical analysis was carried out. “Shapiro Wilks test” was used to evaluate the normality of the data distribution of the study variables. Nonparametric tests were used to determine differences in significance between the comparison groups. Intergroup comparison for the study evaluated variables (burning sensation, pain, lesion score, symptomatic response of burning sensation, symptomatic response of pain, clinical response) and the age difference between the three groups was performed using the “Kruskal-Wallis test”. Intergroup comparison for nominal categorical variables (gender and site) were performed using “chi square test”. Using the “Friedman test” at various time points, an intragroup comparison was made for the research variables that were analyzed. For all comparisons, P 0.05 was considered statistically significant.

## Results

The descriptive statistics pertaining to “demographic data” (mean age group, gender, and site) for both intervention groups showed that the mean age of the 3 groups was 45.60(Group 1), 42.17(Group 2), and 40.25(Group 3). The results on mean age in our study are in accordance with the literature. The male: female ratio of each group was 3 males and 7 females in the control group, and 4 males and 8 females each in study groups 2 and 3. The overall gender distribution showed a female predilection of 23 (67.6%). The buccal mucosa was the most affected region, followed by the buccal mucosa along with the dorsum of the tongue, and the buccal mucosa along with the gingiva. There was no significant difference in the demographic data [mean age group (*p* = 0.71), gender (*p* = 0.87), and site (0.63)] between the intervention groups, indicating that the compared intervention groups were matched samples.

When the research variables were compared between the groups at each of the five time points, the intergroup comparison showed that there was no statistically significant difference between the compared groups (baseline, 14 days, 30 days, 60 days, and 90 days). An intergroup comparison of the study variables (symptomatic reaction of burning sensation (*p* = 0.15), symptomatic response of pain (*p* = 0.70), and clinical response (*p* = 0.89)) showed no statistically significant difference between the compared groups. The intergroup comparison of the variables measured the symptomatic response of burning sensation and pain. Clinical improvement was evaluated as clinical response. Both the parameters were assessed using VAS scale and Thongprasom’s criteria (Tables 2 and 3).

**Table 2:** Intergroup comparison of symptomatic response - burning sensation & pain

	**Groups**	**No burning sensation n(%)**	** + 1 degree imp n(%)**	** + 2 degree imp n(%)**	** + 3 degree imp n(%)**	** + 4 degree imp n(%)**	**Kruskal -wallis test value**	***P* value**
**Symptomatic Response of Burning sensation**	**Group 1**	0(0%)	0(0%)	0(0%)	3(8.8%)	7(20.6%)	3.70	0.15
	**Group 2**	0(0%)	0(0%)	3(8.8%)	5(14.7%)	4(11.8%)		
	**Group 3**	0(0%)	0(0%)	2(5.9%)	3(8.8%)	7(20.6%)		
**Symptomatic Response of Pain**	**Group 1**	2(5.9%)	2(5.9%)	2(5.9%)	3(8.8%)	1(2.9%)	0.68	0.70
	**Group 2**	2(5.9%)	2(5.9%)	4(11.8%)	5(14.7%)	1(2.9%)		
	**Group 3**	0(0%)	1(2.9%)	7(20.6%)	2(5.9%)	2(5.9%)

**Table 3:** Intergroup comparison of clinical response - Lesion score

	**Groups**	**No lesion or normal mucosa (Complete resolution) n (%)**	** + 1 degree imp n (%)**	** + 2 degree imp n (%)**	** + 3 degree imp n (%)**	** + 4 degree imp n (%)**	**-1 degree worsening**	**Kruskal- wallis test value**	***P* value**
**Clinical Response**	**Group 1**	0(0%)	2(5.9%)	6(17.6%)	1(2.9%)	1(2.9%)	0(0%)	0.21	0.89
	**Group 2**	0(0%)	1(2.9%)	9(26.5%)	2(5.9%)	0(0%)	0(0%)		
	**Group 3**	1(2.9%)	1(2.9%)	6(17.6%)	4(11.8%)	0(0%)	0(0%)		

The intragroup comparison of study variables showed a statistically significant difference between the five time periods (baseline, 14 days, 30 days, 60 days, and 90 days) according to the intragroup comparison (*p* = 0.001).

More cases in the “no burning sensation” category were reported at the end of three months for all three groups with percentage distribution of 50%, 33.3%, and 33.3% for groups 1, 2, and 3, respectively (Table 4).

**Table 4:**
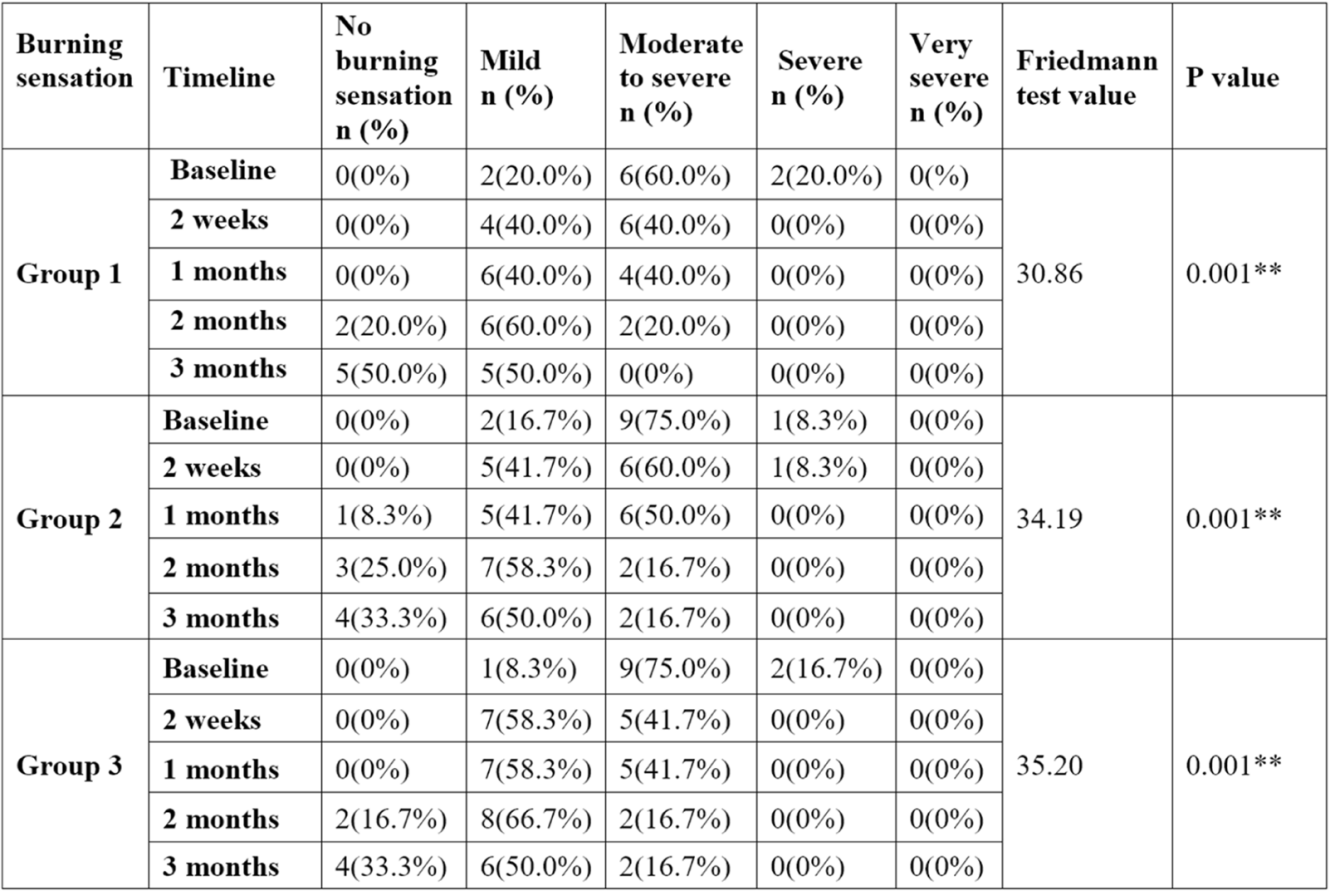
Intragroup comparison of burning sensation at five different time periods

** *p* value is statistically significant

A greater number of cases in the “no pain” category was reported at the end of three months for all three groups with percentage distribution of 90%, 66.7%, and 75% for groups 1, 2, and 3, respectively (Table 5).

**Table 5:**
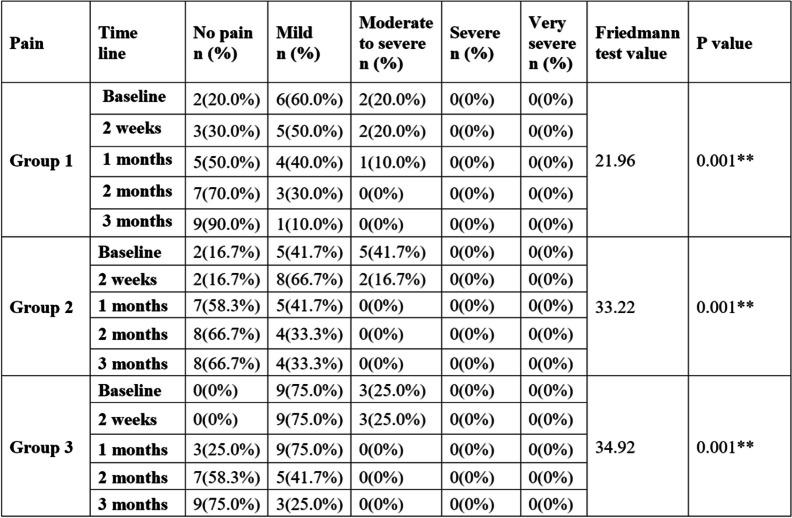
Intragroup comparison of pain at five different time periods

** *p* value is statistically significant

A greater percentage of the absence of lesions was observed at the end of three months for all three groups with percentage distribution of 50%, 41.7%, and 50%, respectively for the compared groups (Table 6).

**Table 6:**
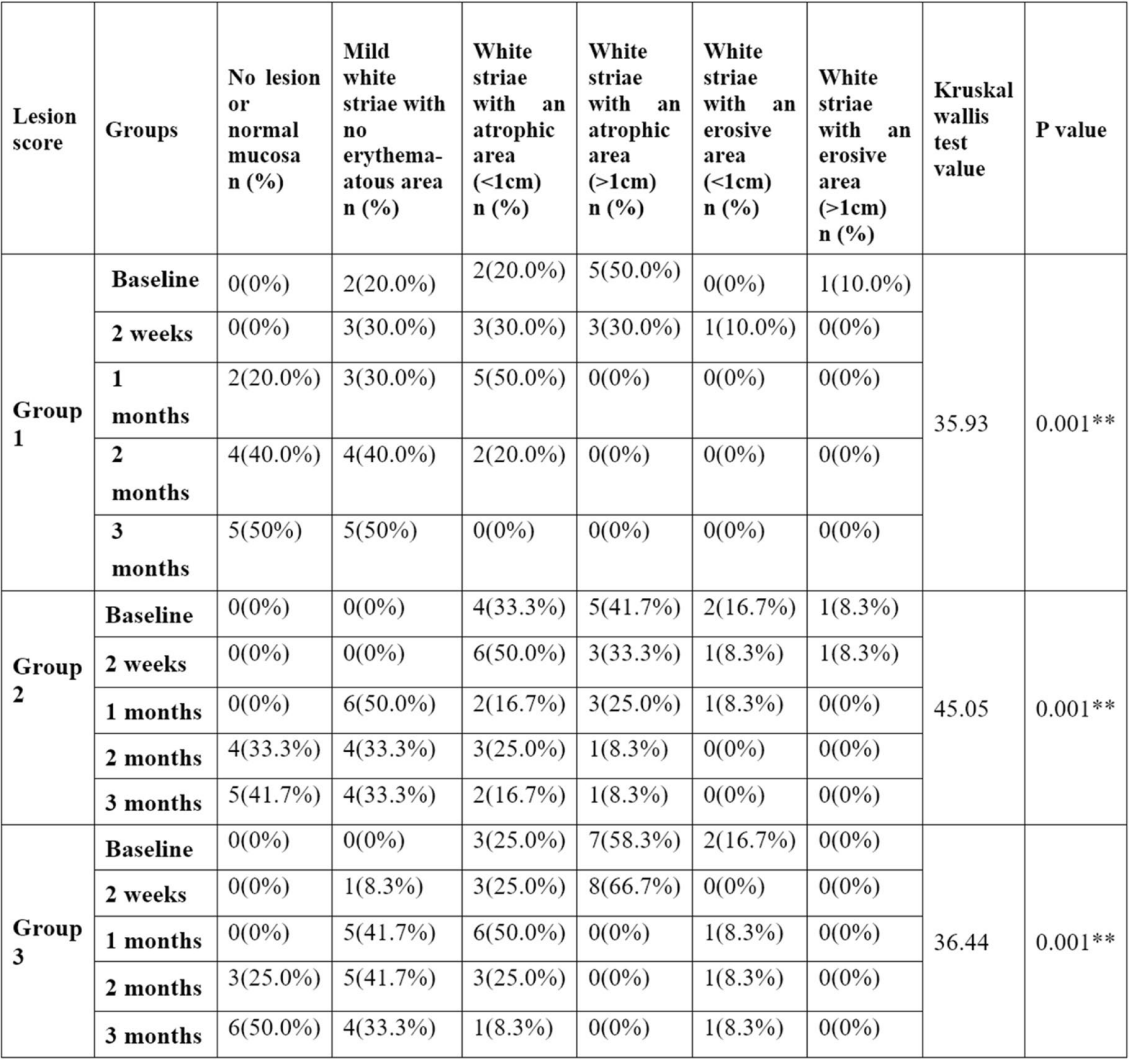
Intragroup comparison of lesion scores at five different time periods

** *p* value is statistically significant

All the participants were followed up for a period of 3 months and there were no dropouts during the study. All the participants in the purslane study group showed partial to complete relief of burning sensation and pain along with partial to complete remission of at the end of three months. No side effects were reported in any of the groups.

## Discussion

PK Mankapure et al., undertook a study to better understand the demographics and clinical characteristics of OLP in 108 patients. The findings revealed that 87.9% of cases involved the buccal mucosa, with females making up 70.4% of those affected [18]. In the study by Bakhshi et al., buccal mucosa involvement was the most prevalent site. In our study, there was a female predilection, and the most frequent site of involvement was the buccal mucosa, which was followed by involvement of both the buccal mucosa and the dorsum of the tongue and both the buccal mucosa and gingiva when compared across all three groups [19].

Kia et al., assessed the comparative efficacy of triamcinolone and curcumin in OLP patients where the use of curcumin showed a significant response. However, few patients had a burning sensation, and few reported the undesirable yellow color of the drug [20]. Choonhakarn et al., compared the efficacy of aloe vera gel with a placebo in treating oral lichen Planus [21]. Nashat et al., assessed the clinical effectiveness of topical 2% chamomile cream with 0.1% triamcinolone acetonide [22].

Najafi et al., in a placebo-controlled clinical trial, systemically treated recurrent aphthous stomatitis with 235 mg purslane, yielding favorable pain control outcomes and reduced recurrence. These results tout the antioxidant and anti-inflammatory efficacy of purslane [23].

In a placebo-controlled study by Agha Hosseini et al., in 2010, oral lichen planus patients were treated with 235 mg capsules prepared from the ethanolic extract of purslane leaves, and the results showed better clinical improvement with no reported side effects making purslane a favorable, safer alternative for treating OLP [11]. The participants were evaluated at five different periods and the clinical improvement was evaluated by calculating the clinical response (CR) and symptomatic response (SR), where 83% of purslane-treated patients showed partial to complete clinical improvement and 17% had no response. A significant decrease in VAS scores was observed. Additionally, the participants treated with purslane did not show any side effects. Similarly, in our study, 41.7% of 5% topical gel-treated participants and 50% of 10% topical gel-treated participants showed complete remission of the clinical lesion, and overall complete relief of burning sensation and pain based on VAS score was observed to be 33.3% for burning sensation at both concentrations and 66.7% and 75% for pain in 5% and 10% concentrations, respectively. Comparing the clinical improvement in both studies, our study showed statistically significant results in the intragroup comparison with complete remission and no recurrence or occurrence of new lesions at the end of three months without any noticeable side effects. Our study participants did not show any adverse consequences, and the use of topical purslane gel at two concentrations showed equivalently significant results compared to 0.1% triamcinolone acetonide thereby advocating purslane as a favorable alternative treatment for OLP.

## Clinical significance

With available literature evidence, our present study is the first of its kind to formulate a topical gel with purslane to treat symptomatic OLP. Our study had a longer follow-up of 3 months compared to other studies in the literature. Additionally, the results were compared as intergroup and intragroup comparisons at all five time periods. Intragroup comparison of all the groups at five different periods showed that both 5% and 10% topical purslane gel showed significant efficacy compared to the gold standard, making it a reliable treatment option.

## Conclusion

Over the period, corticosteroids have been the mainstay treatment for OLP. Undesirable effects of steroids have made it possible for more herbal therapies to be used, and many studies are being focused on in search of safer alternatives. Purslane, a magical herb with a plethora of rich nutrients, easy availability, and a lack of side effects, is beneficial and can be a safer alternative drug in OLP treatment. Future research with larger sample sizes, and longer follow-ups to check recurrences should also be included.

## Data Availability

The datasets generated and/or analyzed during the current study are not publicly available as permission has to be obtained from the institution but are available from the corresponding author on reasonable request.

## Abbreviations

OLP: Oral lichen planus
HI: Herbal intervention
VAS: Visual Analog Scale
SR: Symptomatic Response
CR: Clinical Response
CS: Corticosteroids
